# Enhanced Mathematical Model for Producing Highly Dense Metallic Components through Selective Laser Melting

**DOI:** 10.3390/ma14061571

**Published:** 2021-03-23

**Authors:** Jorge A. Estrada-Díaz, Alex Elías-Zúñiga, Oscar Martínez-Romero, Daniel Olvera-Trejo

**Affiliations:** School of Engineering and Science, Tecnologico de Monterrey, Av. E. Garza Sada 2501 Sur, Monterrey 64849, Mexico; oscar.martinez@tec.mx (O.M.-R.); daniel.olvera.trejo@tec.mx (D.O.-T.)

**Keywords:** selective laser melting, mathematical modeling, manufacturing parameters, densification

## Abstract

**Featured Application:**

**This article provides proper fitting parameters, for a wide variety of metallic alloys, to apply the derived mathematical expression (which allows to determine the scanning speed value needed, with respect to laser power) to produce highly dense components through selective laser melting.**

**Abstract:**

In this work, a previously developed mathematical model to predict bulk density of SLMed (produced via Selective Laser Melting) component is enhanced by taking laser power, scanning speed, hatch spacing, powder’s thermal conductivity and specific heat capacity as independent variables. Experimental data and manufacturing conditions for the selective laser melting (SLM) of metallic materials (which include aluminum, steel, titanium, copper, tungsten and nickel alloys) are adapted from the literature and used to evaluate the validity of the proposed enhanced model. A strong relation between dependent and independent dimensionless products is observed throughout the studied materials. The proposed enhanced mathematical model shows to be highly accurate since the computed root-mean-square-error values (RMSE) does not exceed 5 × 10^−7^. Furthermore, an analytical expression for the prediction of bulk density of SLMed components was developed. From this, an expression for determining the needed scanning speed, with respect to laser power, to achieve highly dense components produced via SLM, is derived.

## 1. Introduction

Selective laser melting (SLM) is an additive manufacturing (AM) process that produces geometrically complex components from metallic powders. It uses a high intensity laser, in an inert atmosphere, to selectively melt specific regions of prelaid powder. The material quickly melts, then cools and solidifies. The final component is built layer-by-layer in an iterative process of powder spreading from a feed container, scanning of the laser and lowering of the build platform [1,2,3,4].

SLM may be seen as a heat transfer process where the laser transfers energy to the powder bed. The powder is melted, and a solidification period is allowed. Conduction, convection and radiation related to heat transfer occurs in the SLM process. When the laser beam hits the powder bed, a fraction of the energy input is absorbed, and the remainder is emitted to the surroundings. Moreover, the absorbed energy causes the material to melt since heat conduction occurs in different directions. Heat conduction takes place from the melt pool to the surrounding powder, from the powder to the substrate, from the substrate to the machine and within the powder itself. Moreover, convective heat flow is also present in the interface of the top layer and the atmosphere in the direction of gas flow [5]. The resulting properties of the piece will depend upon said heat transfer conditions along with subprocesses such as laser systems and optics, energy absorption, phase changes, fluid flow as Marangoni convection, sublimation and ejection of particles [6].

The attainable bulk density of a component produced via SLM is the most important concern. Mechanical properties, and thus component performance, is highly linked to its density [7]. It is sought, then, to obtain pieces as dense as possible (reduce porosity). The adequate selection of process parameters allows obtaining a highly dense components and, thus, excellent mechanical integrity. In this context, dimensional analysis brings huge benefits. By applying Buckingham’s π-theorem, the description of physical problems is considerably eased. It removes unessential information from the regarded problem, reducing the number of variables and, therefore, providing a sharper insight to the essential physical interactions between factors [8]. Physical processes are then not described by dimensional, but rather by non-dimensional quantities. In the same way, the number of required experiments to reveal the complete physical behavior is reduced [9]. Dimensional analysis is based upon Buckingham’s π-theorem, which states that a physical process will be described by *n − k = d* number of dimensionless products, where *n* is the complete set of independent physical quantities influencing the process, and *k* is the chosen dimensionally independent subset (usually the same as the number of fundamental dimensions involved in the problem) [10].

In this work, Buckingham’s π-theorem will be applied, through dimensional analysis, to the multiphysics problem of selective laser melting. In this regard, few have been the published articles that attempt to describe SLM via dimensional analysis. Van Elsen et al. [6] proposed a complete set of independent dimensionless parameters to describe the SLM process. Cardaropoli et al. [11] applied dimensional analysis, defining a set of 16 independent physical quantities, which, according to the authors, influenced SLM the most, arriving at twelve π-products. Khan et al. [12] made a connection between dimensional analysis and SLM in the modeling of the heat source as a function of laser parameters and powder properties. Estrada-Díaz et al. [13] developed a mathematical model, through dimensional analysis, that was able to predict SLMed (produced via Selective Laser Melting) components’ bulk density. Moreover, they developed expressions that enable the user to successfully calculate the needed scanning speed value, with respect to laser power, to achieve highly dense components.

The present article will enhance the work of Estrada et al. by introducing subtle but relevant modifications to the mathematical model and studying its validity on a wide range of metallic materials and alloys. The chosen set of independent physical quantities involved in the dimensional analysis of SLM is modified in this work. The concept of volumetric energy density has been identified by Mishurova et al. [14] and Scipioni et al. [15] not to be a reliable design factor. For this reason, it has been excluded from this new proposed dimensionless model. Additionally, laser power and hatch distance are considered in the model. Thus, a mathematical expression for the prediction of bulk density of metallic pieces produced by selective laser melting is developed. Moreover, through the developed model, we are able to define the needed scanning speed value, with respect to laser power, in order to obtain highly dense metallic components. Thus the experimental data needed for applying our enhanced model focuses on fitting two process parameters that vary depending on the powder material. This work adapts experimental data found in the literature to produce metallic parts via SLM process of materials such as aluminum, steel, nickel, copper, tungsten and titanium metallic alloys. Hence, the material data are used to determine the proper fitting parameters values, which are needed to set the mathematical expressions to be applied by the user to have an efficient SLM fabrication process.

This is a novel approach to the mathematical modeling of SLM. It is of the upmost practical relevance as it provides an easy-to-apply mathematical tool for setting the scanning speed with respect to SLM laser power (or vice versa), to obtain low porosity metallic parts.

In summary, the present article is divided into the following sections: (1) development of a dimensional analysis applying Buckingham’s π-theorem in order to develop a mathematical model for predicting bulk density of SLMed components and for determining the needed scanning speed, with respect to laser power, to attain highly dense components, (2) exploring the validity of the mathematical model through a wide spectrum of metallic materials with the adaption of experimental data from previously published works, (3) calculation of fitting parameters and evaluation of the precision of fit for each referenced work, (5) unification of experimental data for materials in common (AlSi10Mg, Ti6Al4V, In718 and SS316L) for trend analysis and (6) calculation of robust fitting parameters through three different methods for Ti6Al4V, In718 and SS316L. It is important to consider that the specific values of the calculated fitting parameters are valid for the working interval of independent dimensionless product, π_1_. Moreover, heat conductivity and specific heat capacity were taken as constant values.

### Literature Review

Experimental data for the SLM of a wide variety of materials and alloys, such as aluminum, steel, titanium, copper, tungsten and nickel alloys, was adapted from the literature. Their authors, specific material/alloy of work, number of reference, and the identification of the table that contains the corresponding experimental data and manufacturing conditions (thoroughly detailed in Appendix A) are presented in Table 1.

## 2. Methods

The dimensional analysis of the selective laser melting process developed in [13] was revised, and it was decided to exclude the concept of volumetric energy density and incorporate relevant manufacturing parameters like laser power and hatch spacing. The enhanced mathematical model is presented in the following subsection. From dimensional analysis we were able to obtain two relevant findings: (1) a mathematical expression to predict the bulk density of components produced through selective laser melting and (2) a mathematical expression for the calculation of the required scanning speed, with respect to laser power, to attain metallic pieces of low porosity.

The gathered experimental data (Table 1) was used to calculate the dimensionless products *π_0_* and *π_1_*, presented in the following section, to determine the values of the *C* and *α* fitting parameters. The fitting procedure for the work of each author was performed with the aid of MATLAB software (R2020a, 2020, MathWorks, Natick, MA, USA). following the nonlinear least squares method. A robust bisquare fitting was performed to downweigh outliers’ effect on the fit. Similarly, since no coefficient constraints were established, the Levenberg–Marquardt algorithm was implemented. The graphs of dependent, π_0_, and independent, π_1_, dimensionless products alongside the predictions for each referenced work were obtained in order to evaluate the validity of the mathematical model through the spectrum of materials. Likewise, the RMSE (root-mean-square-error) value for each fit was obtained to investigate its precision. 

Then, the collected data was grouped for In718, SS316L, AlSi10Mg and Ti6Al4V, aiming at identifying trends and determining more robust fitting parameters for each material. The determination of the values of *C* and *α* fitting parameters, in this case, was performed following three different methods. The first one has been previously described and will be regarded as method one for future references. The second approach was to consider the average values of *α* from Table 2 for each material and will be regarded as method two. Finally, method three consisted in applying the least squares method, using Microsoft Excel software. The three methods were compared through the calculation of the RMSE value for the respective obtained fit.

**Figure 1:**
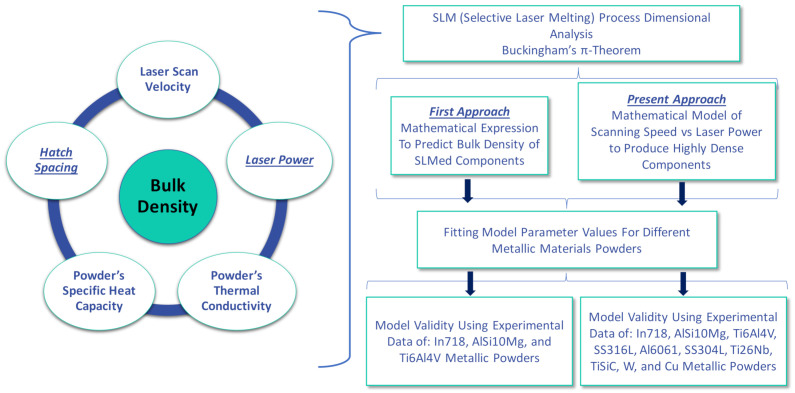
Schematic of the methodology followed throughout the present work.

Figure 1 presents a schematic of the methodology followed in this work. The novelty of the work here presented, in relation to [13] upon which this is improving and expanding, is wide. First of all, the derived mathematical expressions are now of greater practical significance since they now incorporate laser power and hatch spacing, which are directly modifiable relevant manufacturing parameters. Moreover, the validity of the model has been explored through a wide variety of metallic materials. Fitting parameters for each referenced work have also been calculated providing the user with the necessary information to implement the developed expressions and apply them in the actual SLM of the material of choice.

### SLM Dimensional Analysis Development

Laser power and scanning velocity are directly related to the energy that the powder bed will receive. Appropriate combination of both parameters is of the upmost importance as it will ensure proper melt of the powder and bonding between scan tracks and cross-sectional layers. Setting inadequately high laser power and low scanning velocity conditions, leads to powder particle sublimation and ejection from the powder bed. Moreover, it promotes delamination. Likewise, improperly low energetic conditions lead to unmelt powder residues and, thus, an unsuccessful built.

Hatch distance is the separation between laser trajectory lines. It has been found by diverse authors [1,5,14] that greater hatch distance leads to greater porosity and thus, poorer densification. Specific heat capacity is the quantification of energy required to increment a material’s temperature. As the SLM process aims at fully melting the metallic powder, this is a highly relevant parameter. Finally, during the dimensional analysis of SLM process, heat conductivity is assumed to be an independent SLM process parameter in.

Following the work presented by Estrada et al. [13], a subtle modification to the set of independent variables is introduced. Volumetric energy density is no longer regarded as an independent physical quantity since it has been identified not to be a reliable design factor for the SLM process [14,15]. On the other hand, laser power (*P*) and hatch spacing (*h*) will be incorporated in the dimensional analysis. They, alongside scanning speed (*v*), specific heat capacity (*C_p_*) and heat conductivity (*κ*) will be held as the complete set of independent physical quantities that most influence the densification (*ρ*) of SLMed components, as Equation (1).

(1) $$\rho =\;f\left(P,h,v,C_{p},\;\kappa \right)\;\;\;\;$$

By applying Buckingham’s π-theorem and the proper dimensional analysis procedure, the dependent and independent dimensionless products are obtained, and presented in Equations (2) and (3) respectively.

(2) $$\pi_{0}=\;\frac{\rho \;v^{3}\;h^{2}}{P}$$

(3) $$\pi_{1}=\;\frac{\kappa \;v^{2}\;h}{P\;C_{p}}$$

The causal relationship form of dimensional analysis is presented in Equation (4). This states that the dependent dimensionless product *π*_0_ will be a function of the independent one *π*_1_.

(4) $$\pi_{0}=f\left(\pi_{1}\right)$$

Assuming a power law behavior between dependent and independent dimensionless products, Equation (5) is derived, where *C* and *α* are fitting parameters to be determined through experimentation. In this work, instead of turning to new experimentation, previously published experimental data has been adapted and used for the determination of the specific numerical values of the fitting parameters.

(5) $$\pi_{0}=C\pi_{1}^{\alpha }$$

Substituting the definitions for dependent (*π*_0_) and independent (*π*_1_) dimensionless numbers, in Equations (2) and (3) respectively, into Equation (5), and solving for bulk density, the expression to predict the SLMed components’ bulk density is obtained and presented in Equation (6).

(6) $$\rho =C\;\frac{P}{v^{3}h^{2}}\;{\left(\frac{\kappa \;v^{2}\;h}{P\;C_{p}}\right)}^{\alpha }$$

Solving Equation (6) for scanning speed, *v*, and using the value of theoretical density *ρ_th_* defined for each material, an expression for determining the needed scanning speed, with respect to laser power, to obtain highly dense AM components is obtained, and presented in Equation (7).

(7) $$v={\left(P^{1-\alpha }\frac{C}{\rho_{th}h^{2}}{\left(\frac{\kappa h}{C_{p}}\right)}^{\alpha }\right)}^{\frac{1}{3-2\alpha }}$$

## 3. Results

### Calculation of C and α Fitting Parameters for Multiple Metallic Alloys

For each work referenced, the dependent (*π*_0_) and independent (*π*_1_) dimensionless products were calculated using Equations (2) and (3) respectively. The adapted experimental data (*ρ*), manufacturing conditions (*P*, *v* and *h*) and material properties (*κ* and *C_p_*) used to perform these calculations are contained in Appendix A. The specific table identifier for each reference is contained in Table 1. The calculated *C* and *α* fitting parameters values, calculated with method one, in the respective independent dimensionless product range *π*_1_ of validity, are presented in Table 2. The work from where experimental data was adapted, the material where the study was performed and the root mean square error (RMSE) value for each fit are presented as well in Table 2. 

**Figure 2:**
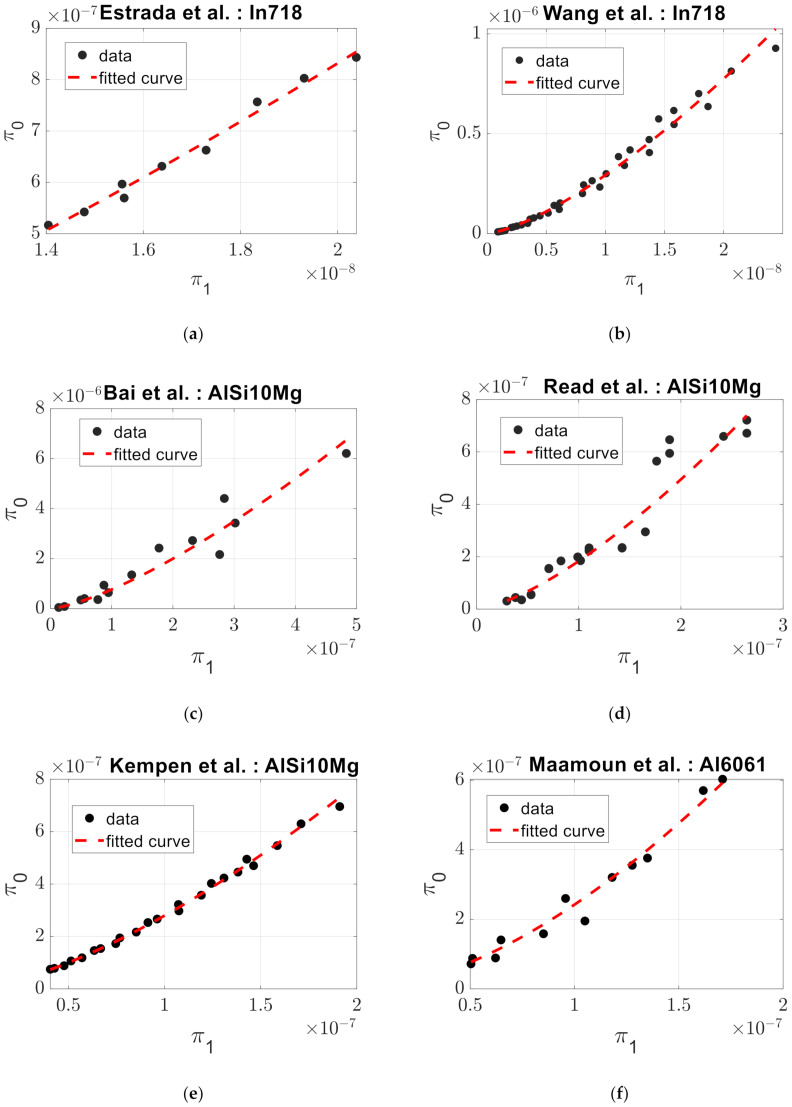

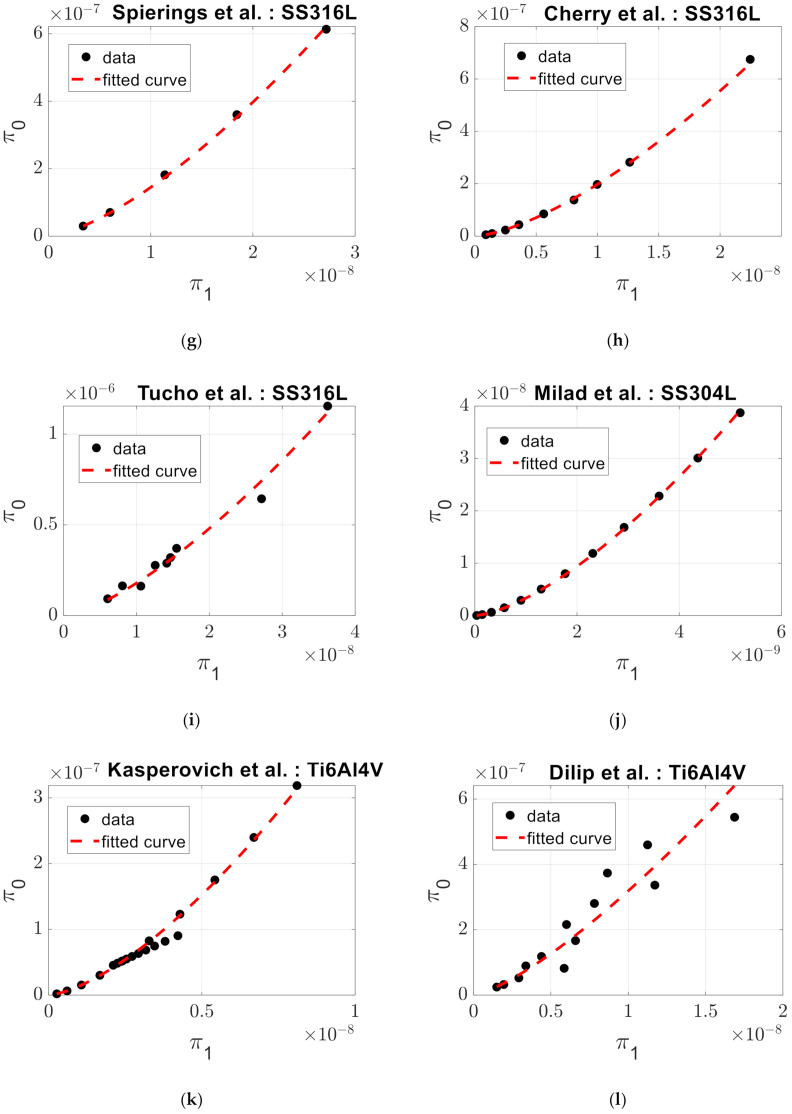

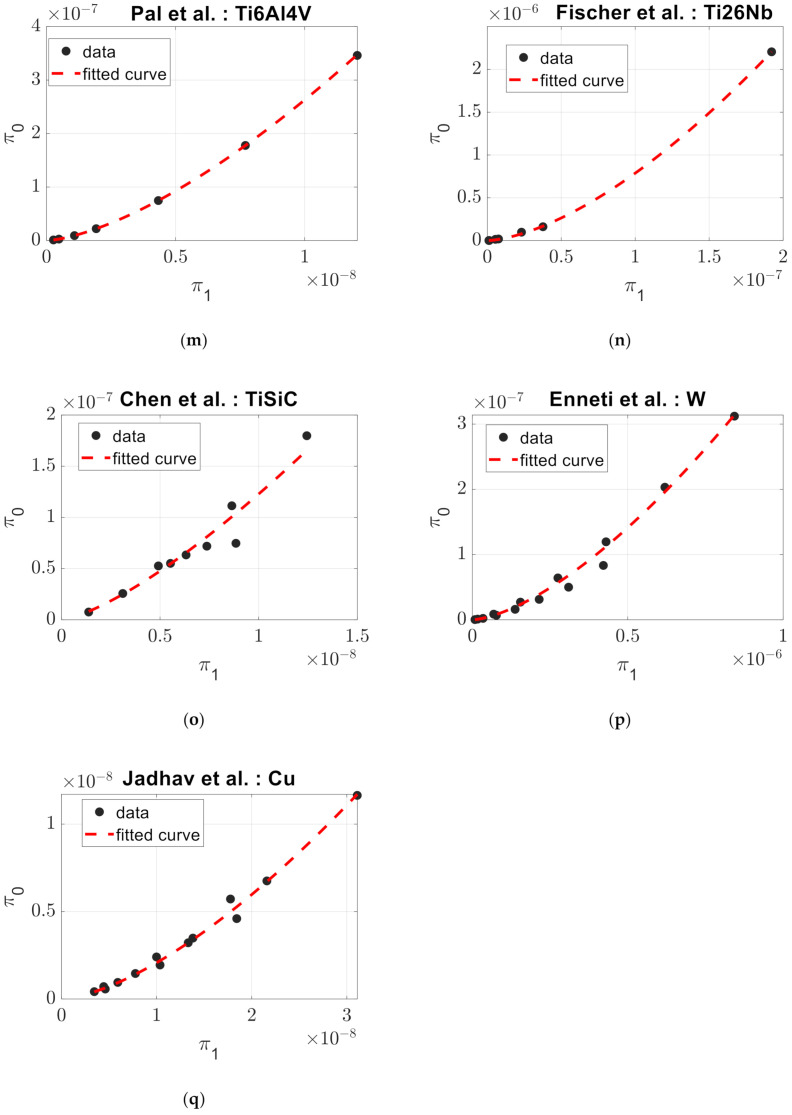
Computed curves of dependent (*π*_0_) versus independent (*π*_1_) dimensionless products fitted for different materials used to produce metallic components with the selective laser melting (SLM) process: (**a**) Estrada et al. [13] with In718, (**b**) Wang et al. [16] with In718, (**c**) Bai et al. [17] with AlSi10Mg, (**d**) Read et al. [18] with AlSi10Mg, (**e**) Kempen et al. [19] with AlSi10Mg, (**f**) Maamoun et al. [20] with Al6061, (**g**) Spierings et al. [21] with SS316L, (**h**) Cherry et al. [22] with SS316L, (**i**) Tucho et al. [23] with SS316L, (**j**) Milad et al. [24] with SS304L, (**k**) Kasperovich et al. [25] with Ti6Al4V, (**l**) Dilip et al. [26] with Ti6Al4V, (**m**) Pal et al. [27] with Ti6Al4V, (**n**) Fischer et al. [28] with Ti26Nb, (**o**) Chen et al. [29] with TiSiC, (**p**) Enneti et al. [30] with W and (**q**) Jadhav et al. [31] with Cu. A strong relation between dependent and independent dimensionless products is observed in all materials, evidencing the validity of the developed model. Moreover, the prediction is of high precision.

Figure 2 presents the experimental data points for the dependent (*π*_0_) and independent (*π*_1_) dimensionless products calculated with Equations (2) and (3) respectively. Moreover, it includes the prediction of the dependent dimensionless product (*π*_0_). This line is drawn using Equation (5) by incorporating the independent dimensionless product (*π*_1_), defined in Equation (3), alongside the respective *C* and *α* fitting parameters values for each work, presented in Table 2.

**Figure 3:**
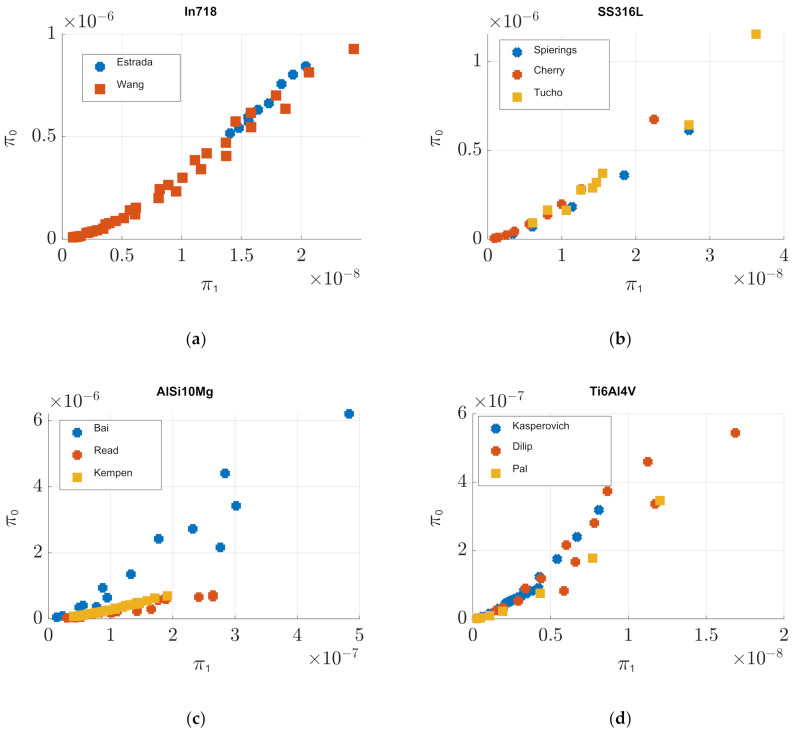
Computed curves of dependent (*π*_0_) vs. independent (*π*_1_) dimensionless products plotted by plugging the adapted experimental data, manufacturing conditions and relevant physical properties of each material contained in the following tables: (**a**) In718 with data from Table A1 and Table A2, adapted from [13,16], (**b**) SS316L with data from Table A7, Table A8 and Table A9, adapted from [21,22,23], (**c**) AlSi10Mg with data from Table A3, Table A4 and Table A5, adapted from [17,18,19] and (**d**) Ti6Al4V with data from Table A11, Table A12 and Table A13, adapted from [25,26,27]. A clear cohesive trend is observed on In718, SS316L and Ti6Al4V. However, that is not the case for AlSi10Mg. For AlSi10Mg, the three different powders used by each author possess considerably different Si content. This is related to solidification and phase change in the selective laser melting process. Another potential source of discrepancies consists of the inability to compare powders’ morphology or particle size distribution.

Figure 3 presents the graphs of dependent (*π*_0_) vs. independent (*π*_1_) dimensionless products for different materials. Both were calculated by plugging the adapted experimental data, manufacturing conditions and material properties (presented in Appendix A), into Equations (2) and (3). Figure 3a was constructed by considering data from Table A1 and Table A2, for In718. Figure 3b, for SS316L, used the contents of Table A7, Table A8 and Table A9. Table A3, Table A4 and Table A5 were used for the drawing of Figure 3c, for AlSi10Mg. Finally, Figure 3d was generated with the information presented in Table A11, Table A12 and Table A13, for Ti6Al4V.

The numerical values of the *C* and *α* fitting parameters for In718, SS316L and Ti6Al4V, from Equation (5), were calculated following the three different methods discussed in the previous section and are presented in Table 3. A mismatch in the observed behavior of π_0_ with respect to π_1_ for the referenced works of AlSi10Mg, in Figure 2c, was observed. For this reason, fitting parameters were not calculated for this material and were thus not included in Table 3. Instead, potential sources of this discrepancy were explored in the following section.

**Figure 4:**
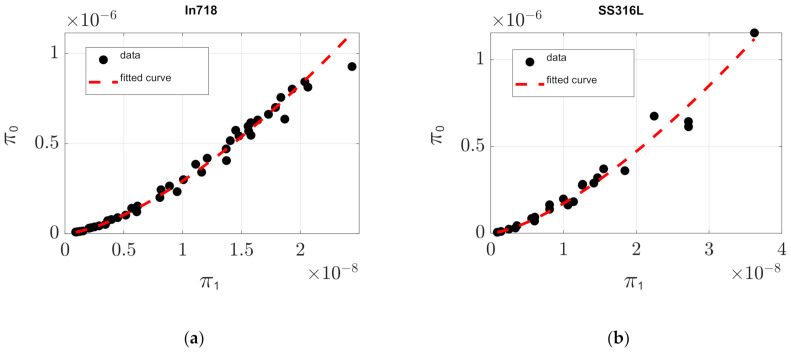

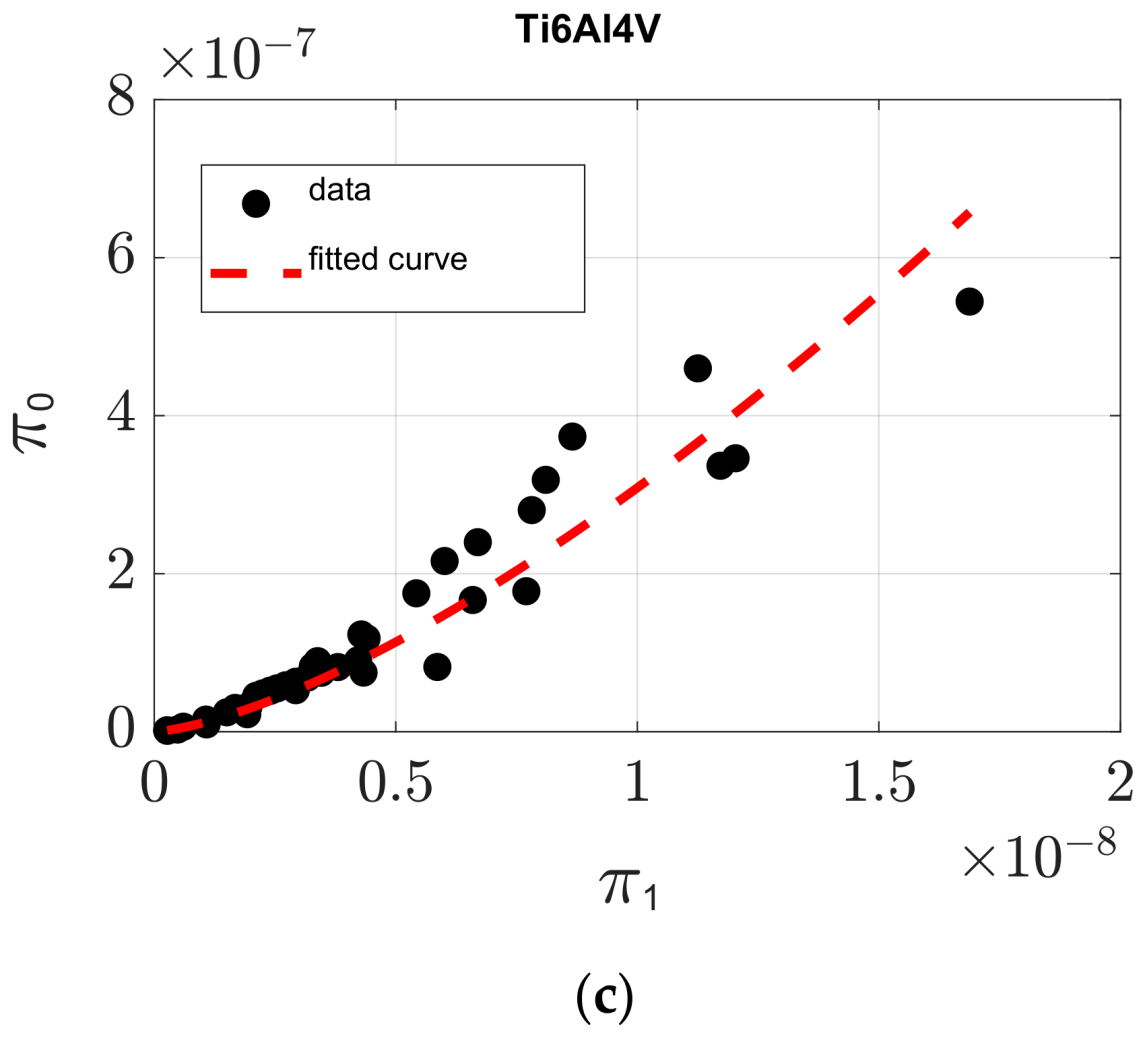
Computed curves of dependent (*π*_0_) vs. independent (*π*_1_) dimensionless products with experimental data collected from: (**a**) In718, (**b**) SS316L and (**c**) Ti6Al4V. A strong relation between dimensionless products is observed. These plots conform the proposed model prediction.

In Figure 4, the dependent (*π*_0_) vs. independent (*π*_1_) dimensionless products graphs with the adapted experimental data alongside its prediction is presented, incorporating the proper C and α values just derived, for In718 (Figure 4a), SS316L (Figure 4b) and Ti6Al4V (Figure 4c). 

## 4. Discussion

The dimensional analysis provided an insight on the interaction of manufacturing parameters and physical properties involved in the SLM process. From Equation (6), we could conclude that the bulk density of an AM component produced via SLM will be positively influenced by laser power supply, and inversely by scanning speed and hatch distance. These conclusions are physically consistent. With higher laser power, more energy is striking the powder bed and, thus, it is easier to achieve complete melt. In contrast, with high scanning speed the laser strikes within a very short time period the powder bed. Consequently, heating of the material was negatively affected. Additionally, greater hatch distance will lead to poorer densification as weld lines are more spaced out, promoting the appearance of inner defects.

An enhanced mathematical model to determine the scanning speed as a function of the specific heat capacity, heat conductivity, laser power and hatching spacing was derived. Therefore, Equation (7) can be used to produce highly dense metallic parts via the SLM process. It is evident that using the proposed mathematical model given by Equation (7) can assist the user to improve the quality of the fabricated parts while reducing material waste and tuning machine settings time.

The developed mathematical model was applied to a wide variety of metallic alloys varying from nickel, aluminum, stainless steel, titanium, copper and tungsten alloys. Relevant powders’ physical properties, manufacturing conditions and experimental data, contained in Appendix A, were used to calculate *π*_0_ and *π*_1_ from Equations (2) and (3). Then, *C* and *α* values were calculated and presented in Table 2. Figure 3 illustrates the experimental data points for *π*_0_ and *π*_1_ along with the prediction for *π*_0_, given by Equation (5), which incorporates the calculated *C* and *α* values. From this graph, it was observed that there is a strong correlation between dimensionless products for all materials, proving that the developed dimensional analysis has been successful and that it remains valid independent of the material at hand. We were dealing with a model of high precision since the RMSE values, in Table 2, for each fit, were quite low. In this case, the RMSE value ranged from 1.9104 × 10^−10^ to 5.0408 × 10^−7^. The calculated values of *α* for the studied materials ranged from 1.335 to 1.667. This reflects that selective laser melting was a non-linear process with respect to *π*_1_. 

It is observed from Figure 3a that for In718, there is great agreement between the works of Estrada et al. [13] and Wang et al. [16] since the trends for the responses of the dependent dimensionless product with respect to the independent one are similar. The same is observed for SS316L, in Figure 3b, between the works of Spiering et al. [21], Cherry et al. [22] and Tucho et al. [23]. For Ti6Al4V, there was also a clear, easily recognizable, trend, which is congruent throughout the works of Kasperovich et al. [25], Dilip et al. [26] and Pal et al. [27]. From Table 3, we can observe that the best result for In718 was obtained using fitting method three, as the RMSE was the lowest found, with values of *C* = 285,400 and *α* = 1.499 and RMSE = 2.5790 × 10^−8^. For SS316L, the best fit was obtained following method two, with values of *C* = 276,830 and *α* = 1.456 and RMSE = 4.2349 × 10^−8^. For Ti6Al4V, the best fit was achieved for the values of *C* = 110,600, *α* = 1.444 and RMSE = 2.7403 × 10^−8^. Figure 3 reflects that the enhanced mathematical model and calculated fitting parameters are of high precision.

For the AlSi10Mg produced metallic samples shown in Figure 3c, some discrepancies were observed in the behavior of π_0_ versus π_1_ due to the material’s chemical composition. Table 4 summarizes the chemical composition of the AlSi10Mg powder used by Bai et al. [17], Read et al. [18] and Kempen et al. [19]. Silicon content in each one varied considerably. Silicon content in Al-Si alloys is related to solidification in selective laser melting. Lower contents of silicon lead to lesser absorption of the energy provided by the laser. Moreover, it causes a larger temperature difference between the solidus and liquidus lines influencing the phase change phenomenon [32,33]. Powder morphology has been identified to be of high importance in the selective laser melting process. Read et al. and Kempen et al. provide a scanning electron microscopy (SEM) image of the AlSi10Mg powder used. Both are of similar shape and size distribution. However, Bai et al. did not. Thus, powder morphology was identified as a possible source for the unexpected behavior observed in the trends of the dependent dimensionless product, π_0_, in AlSi10Mg. 

## 5. Conclusions

In this article, an enhanced dimensional analysis model for the bulk density of SLMed components was developed. A general expression capable of predicting the bulk density of metallic components produced by selective laser melting was derived. The relevance of the enhanced mathematical expression was validated with a wide variety of materials proving that it accurately describes the physical process of SLM and that remained valid throughout a broad spectrum of metallic materials, which include aluminum, steel, titanium, copper, tungsten and nickel alloys.

A general expression, given by Equation (7), for determining the scanning speed needed, with respect to laser power, to achieve highly dense components built by the SLM process, was derived applying the dimensional analysis and Buckingham’s π-theorem. By incorporating the *C* and *α* values presented in Table 2 and Table 3 into Equation (6), it was possible to predict the resulting bulk density of the SLMed component. By substituting into Equation (7) the theoretical density value for the material, the user is now able to determine the needed scanning speed value, with respect to the laser power supply, to achieve SLMed components of high densification.

Applying the enhanced mathematical model provides a tool for the proper tuning of manufacturing parameters to produce highly dense final components through selective laser melting, with the potential to bring great economic and resource-saving benefits. The work here presented is of great practical relevance since it elucidates information and mathematical expressions needed to produce components of low porosity, and high mechanical integrity from a wide variety of metallic materials, via selective laser melting. 

The present work expands upon the implementation of dimensional analysis to model the complex process of selective laser melting. Future research on the field should explore the applicability of Buckingham’s π-theorem to investigate properties and characteristics of interest and high relevance in selective laser melting using advanced material metallic powders. Moreover, this may be translated not only to powder bed fusion technologies (category of additive manufacturing to which SLM belongs to) but also to different additive manufacturing processes, e.g., fused deposition modeling. Further implementation of the developed mathematical expressions may be focused on the construction of components with a porosity value of choice for applications where needed. 

## Figures and Tables

**Table 1:** Authors, material, reference and ID of table containing the adapted experimental data in Appendix A.

Authors	Material	Reference	ID of Table Containing the Experimental Data
Estrada et al.	In718	[13]	A1
Wang et al.		[16]	A2
Bai et al.	AlSi10Mg	[17]	A3
Read et al.		[18]	A4
Kempen et al.		[19]	A5
Maamoun et al.	Al6061	[20]	A6
Spierings et al.	SS316L	[21]	A7
Cherry et al.		[22]	A8
Tucho et al.		[23]	A9
Milad et al.	SS304L	[24]	A10
Kasperovich et al.	Ti6Al4V	[25]	A11
Dilip et al.		[26]	A12
Pal et al.		[27]	A13
Fischer et al.	Ti26Nb	[28]	A14
Chen et al.	TiSiC	[29]	A15
Enneti et al.	W	[30]	A16
Jadhav et al.	Cu	[31]	A17

**Table 2:** *C* and *α* fitting parameters values, calculated with method one, in the respective independent dimensionless product range *π*_1_ of validity.

Reference	Material	*C*	*α*	*π*_1_ Range	RMSE
[13]	In718	44,370	1.393	1.40 × 10^−8^–2.04 × 10^−8^	1.5678 × 10^−8^
[16]		50,560	1.405	9.07 × 10^−10^–2.44 × 10^−8^	2.5474 × 10^−8^
[17]	AlSi10Mg	3418	1.378	1.34 × 10^−8^–4.83 × 10^−7^	5.0408 × 10^−7^
[18]		1956	1.433	2.96 × 10^−8^–2.65 × 10^−7^	4.4869 × 10^−8^
[19]		6783	1.484	4.06 × 10^−8^–1.91 × 10^−7^	1.1016 × 10^−8^
[20]	Al6061	113,000	1.667	5.04 × 10^−8^–1.71 × 10^−7^	3.1068 × 10^−8^
[21]	SS316L	66,370	1.458	3.39 × 10^−9^–2.72 × 10^−8^	9.0529 × 10^−9^
[22]		156,600	1.487	8.98 × 10^−10^–2.25 × 10^−8^	3.3363 × 10^−9^
[23]		44,200	1.424	6.05 × 10^−9^–3.62 × 10^−8^	4.9775 × 10^−8^
[24]	SS304L	95,400	1.495	3.61 × 10^−11^–5.19 × 10^−9^	1.9104 × 10^−10^
[25]	Ti6Al4V	395,400	1.495	2.68 × 10^−10^–8.10 × 10^−9^	5.9149 × 10^−9^
[26]		15,370	1.335	1.50 × 10^−9^–1.69 × 10^−8^	6.9473 × 10^−8^
[27]		275,700	1.503	2.71 × 10^−10^–1.20 × 10^−8^	2.1739 × 10^−10^
[28]	Ti26Nb	85,540	1.577	1.10 × 10^−9^–1.92 × 10^−7^	1.0085 × 10^−8^
[29]	TiSiC	10,440	1.366	1.38 × 10^−9^–1.24 × 10^−8^	1.4291 × 10^−8^
[30]	W	598.8	1.528	8.61 × 10^−9^–8.43 × 10^−7^	8.5970 × 10^−9^
[31]	Cu	3300	1.525	3.46 × 10^−9^–3.11 × 10^−8^	2.1069 × 10^−10^

**Table 3:** Fitting parameters calculation for In718, SS316L and Ti6Al4V.

Material	Method	C	α	RMSE
In718	One	69,960	1.42	2.6571 × 10^−8^
	Two	47,840	1.399	2.6626 × 10^−8^
	Three	285,400	1.499	2.5790 × 10^−8^
SS316L	One	74,260	1.454	4.2401 × 10^−8^
	Two	76,820	1.456	4.2349 × 10^−8^
	Three	67,190	1.449	4.2980 × 10^−8^
Ti6Al4V	One	5456,000	1.655	4.696 × 10^−8^
	Two	110,600	1.444	2.7403 × 10^−8^
	Three	186,500	1.473	3.0476 × 10^−8^

**Table 4:** Chemical composition of AlSi10Mg powders used in Bai et al. [17], Read et al. [18] and Kempen et al. [19].

Reference	Element wt %										
	Al	Si	Fe	Cu	Mn	Mg	Zn	Ni	Pb	Sn	Ti
Bai et al. [17]	88.73	10.6	0.19	0.02	-	0.45	<0.01	-	-	-	-
Read et al. [18]	89.585	9.92	0.137	-	0.004	0.291	0.01	0.04	0.004	0.003	0.006
Kempen et al. [19]	90.38	9.02	0.123	0.006	-	0.471	-	-	-	-	-

## Data Availability

Data available on request due to restrictions, e.g., privacy. The data presented in this study are available on request from the corresponding author.

## Appendix A

This appendix contains the tables of relevant physical properties, manufacturing parameters and experimental data adapted from each cited work.

**Table A1:** Manufacturing parameters and experimental data adapted from [13]. Complementing relevant information and assumptions are the following; material: In718, theoretical density *ρ_th_* = 8190 kg/m^3^, heat conductivity *κ* = 11.4 W/mK and specific heat capacity *C_p_* = 435 J/kgK.

ID	*P* (W)	*v* (m/s)	*h* (μm)	Relative Density	Bulk Density (kg/m^3^)
1	360	2	70	0.9457	7745.66
2	360	1.84	70	0.9510	7788.83
3	360	1.75	70	0.9532	7807.09
4	380	2	70	0.9502	7781.87
5	380	1.84	70	0.9564	7832.60
6	380	1.75	70	0.9583	7848.19
7	400	2	70	0.9427	7721.01
8	400	1.84	70	0.9513	7791.15
9	400	1.75	70	0.9608	7869.09

**Table A2:** Manufacturing parameters and experimental data adapted from [16]. Complementing relevant information and assumptions are the following; material: In718, theoretical density *ρ_th_* = 8190 kg/m^3^, heat conductivity *κ* = 11.4 W/mK and specific heat capacity *C_p_* = 435 J/kgK.

ID	*P* (W)	*v* (m/s)	*h* (μm)	Relative Density	Bulk Density (kg/m^3^)
1	370	0.4	80	0.9643	7897.62
2	370	0.6	80	0.9857	8072.88
3	370	0.8	80	0.9968	8163.79
4	370	1	80	0.9949	8148.23
5	370	1.2	80	0.9964	8160.52
6	370	1.4	80	0.9915	8120.39
7	370	1.6	80	0.9897	8105.64
8	340	0.4	80	0.9647	7900.89
9	340	0.6	80	0.9948	8147.41
10	340	0.8	80	0.9934	8135.95
11	340	1	80	0.9940	8140.86
12	340	1.2	80	0.9930	8132.67
13	340	1.4	80	0.9903	8110.56
14	340	1.6	80	0.9760	7993.44
15	300	0.4	80	0.9690	7936.11
16	300	0.6	80	0.9963	8159.70
17	300	0.8	80	0.9937	8138.40
18	300	1.2	80	0.9928	8131.03
19	300	1.4	80	0.9817	8040.12
20	300	1.6	80	0.9794	8021.29
21	260	0.4	80	0.9756	7990.16
22	260	0.6	80	0.9966	8162.15
23	260	0.8	80	0.9956	8153.96
24	260	1	80	0.9945	8144.96
25	260	1.2	80	0.9798	8024.56
26	260	1.4	80	0.9876	8088.44
27	260	1.6	80	0.9854	8070.43
28	220	0.4	80	0.9846	8063.87
29	220	0.6	80	0.9943	8143.32
30	220	0.8	80	0.9932	8134.31
31	220	1	80	0.9780	8009.82
32	220	1.2	80	0.9855	8071.25
33	220	1.4	80	0.9730	7968.87
34	220	1.6	80	0.9509	7787.87

**Table A3:** Manufacturing parameters and experimental data adapted from [17]. Complementing relevant information and assumptions are the following; material: AlSi10Mg, theoretical density *ρ_th_* = 2680 kg/m^3^, heat conductivity *κ* = 110 W/mK and specific heat capacity *C_p_* = 910 J/kgK.

ID	*P* (W)	*v* (m/s)	*h* (μm)	Relative Density	Bulk Density (kg/m^3^)
1	625	1.4	350	0.9382	2514.38
2	950	1.4	350	0.9915	2657.22
3	788	2.3	350	0.8696	2330.53
4	463	2.3	350	0.7198	1929.06
5	300	1.4	350	0.7213	1933.08
6	463	0.5	350	0.9957	2668.48
7	788	0.5	350	0.9448	2532.06
8	788	1.7	400	0.9067	2429.96
9	463	1.7	400	0.7527	2017.24
10	625	0.8	400	0.9916	2657.49
11	788	1.099	300	0.9977	2673.84
12	463	1.099	300	0.9292	2490.26
13	625	2	300	0.8830	2366.44
14	300	0.8	300	0.8837	2368.32

**Table A4:** Manufacturing parameters and experimental data adapted from [18]. Complementing relevant information and assumptions are the following; material: AlSi10Mg, theoretical density *ρ_th_* = 2680 kg/m^3^, heat conductivity *κ* = 110 W/mK and specific heat capacity *C_p_* = 910 J/kgK.

ID	*P* (W)	*v* (m/s)	*h* (μm)	Relative Density	Bulk Density (kg/m^3^)
1	125	1.675	52.5	0.8390	2248.52
2	125	1.675	97.5	0.7530	2018.04
3	125	1.025	97.5	0.9060	2428.08
4	150	1.35	120	0.8920	2390.56
5	125	1.675	97.5	0.7010	1878.68
6	150	0.7	75	0.8960	2401.28
7	150	1.35	75	0.9010	2414.68
8	125	1.675	52.5	0.8460	2267.28
9	175	1.025	97.5	0.9830	2634.44
10	175	1.675	97.5	0.9450	2532.60
11	125	1.025	52.5	0.8820	2363.76
12	150	1.35	30	0.8950	2398.60
13	125	1.025	52.5	0.8590	2302.12
14	150	1.35	75	0.9250	2479.00
15	100	1.35	75	0.7950	2130.60
16	150	1.35	75	0.8990	2409.32
17	175	1.025	52.5	0.9650	2586.20
18	125	1.025	97.5	0.9070	2430.76
19	175	1.675	52.5	0.9360	2508.48
20	175	1.675	97.5	0.8690	2328.92
21	200	1.35	75	0.9920	2658.56
22	150	2	75	0.8200	2197.60
23	175	1.675	52.5	0.9320	2497.76
24	150	1.35	75	0.9450	2532.60
25	150	1.35	75	0.9270	2484.36
26	175	1.025	97.5	0.9920	2658.56
27	175	1.025	52.5	0.9760	2615.68

**Table A5:** Manufacturing parameters and experimental data adapted from [19]. Complementing relevant information and assumptions are the following; material: AlSi10Mg, theoretical density *ρ_th_* = 2680 kg/m^3^, heat conductivity *κ* = 110 W/mK and specific heat capacity *C_p_* = 910 J/kgK.

ID	*P* (W)	*v* (m/s)	*h* (μm)	Relative Density	Bulk Density (kg/m^3^)
1	190	0.8	105	0.9835	2635.73
2	190	1	105	0.9887	2649.58
3	190	1.2	105	0.9915	2657.09
4	190	1.4	105	0.9913	2656.60
5	190	1.6	105	0.9887	2649.80
6	180	0.9	105	0.9902	2653.84
7	180	1.1	105	0.9915	2657.33
8	180	1.3	105	0.9910	2655.75
9	180	1.4	105	0.9891	2650.81
10	180	1.5	105	0.9868	2644.54
11	170	0.8	105	0.9907	2655.05
12	170	1	105	0.9931	2661.51
13	170	1.2	105	0.9907	2655.02
14	170	1.4	105	0.9853	2640.60
15	170	1.6	105	0.9772	2618.79
16	200	0.8	105	0.9883	2648.54
17	200	0.9	105	0.9882	2648.40
18	200	1	105	0.9905	2654.43
19	200	1.1	105	0.9887	2649.80
20	200	1.2	105	0.9911	2656.15
21	200	1.3	105	0.9916	2657.46
22	200	1.4	105	0.9925	2659.85
23	200	1.5	105	0.9924	2659.63

**Table A6:** Manufacturing parameters and experimental data, adapted from [20]. Complementing relevant information and assumptions are the following; material: Al6061, theoretical density *ρ_th_* = 2700 kg/m^3^, heat conductivity *κ* = 167 W/mK and specific heat capacity *C_p_* = 896 J/kgK.

ID	*P* (W)	*v* (m/s)	*h* (μm)	Relative Density	Bulk Density (kg/m^3^)
1	350	1.3	190	0.9852	2659.96
2	370	1.3	190	0.9852	2659.96
3	300	1	190	0.9863	2662.96
4	350	1.3	150	0.9859	2661.96
5	370	1.3	150	0.9848	2658.96
6	370	1	190	0.9867	2663.96
7	350	0.8	190	0.9863	2662.96
8	300	1.3	100	0.9867	2663.98
9	370	1.3	100	0.9874	2665.93
10	350	0.8	150	0.9859	2661.98
11	300	1	100	0.9867	2663.98
12	370	1	100	0.9855	2660.96

**Table A7:** Manufacturing parameters and experimental data adapted from [21]. Complementing relevant information and assumptions are the following; material: SS316L, theoretical density *ρ_th_* = 8000 kg/m^3^, heat conductivity *κ* = 16.3 W/mK and specific heat capacity *C_p_* = 500 J/kgK.

ID	*P* (W)	*v* (m/s)	*h* (μm)	Relative Density	Bulk Density (kg/m^3^)
1	104	0.3	120	0.993	7944
2	104	0.4	120	0.990	7920
3	104	0.55	120	0.986	7888
4	104	0.7	120	0.948	7584
5	104	0.85	120	0.902	7216

**Table A8:** Manufacturing parameters and experimental data adapted from [22]. Complementing relevant information and assumptions are the following; material: SS316L, theoretical density *ρ_th_* = 8000 kg/m^3^, heat conductivity *κ* = 16.3 W/mK and specific heat capacity *C_p_* = 500 J/kgK.

ID	*P* (W)	*v* (m/s)	*h* (μm)	Relative Density	Bulk Density (kg/m^3^)
1	180	0.33	124	0.9113	7290.56
2	180	0.67	124	0.9741	7792.80
3	180	1.00	124	0.9875	7900.16
4	180	0.25	124	0.9233	7386.16
5	180	0.50	124	0.9923	7938.56
6	180	0.75	124	0.9782	7825.76
7	180	0.20	124	0.9629	7702.80
8	180	0.40	124	0.9965	7971.68
9	180	0.60	124	0.9346	7476.40

**Table A9:** Manufacturing parameters and experimental data adapted from [23]. Complementing relevant information and assumptions are the following; material: SS316L, theoretical density *ρ_th_* = 8000 kg/m^3^, heat conductivity *κ* = 16.3 W/mK and specific heat capacity *C_p_* = 500 J/kgK.

ID	*P* (W)	*v* (m/s)	*h* (μm)	Relative Density	Bulk Density (kg/m^3^)
1	150	1.25	80	0.9657	7725.6
2	200	1.667	80	0.9738	7790.4
3	150	0.714	140	0.9746	7796.8
4	150	0.75	120	0.9872	7897.6
5	175	0.75	120	0.9973	7978.4
6	150	0.781	80	0.9986	7988.8
7	200	1.042	80	0.997	7976.0
8	150	0.446	140	0.9984	7987.2
9	200	0.595	140	0.9927	7941.6

**Table A10:** Manufacturing parameters and experimental data adapted from [24]. Complementing relevant information and assumptions are the following; material: SS304L, theoretical density *ρ_th_* = 8000 kg/m^3^, heat conductivity *κ* = 15.2 W/mK and specific heat capacity *C_p_* = 500 J/kgK.

ID	*P* (W)	*v* (m/s)	*h* (μm)	Relative Density	Bulk Density (kg/m^3^)
1	105	0.05	50	0.9915	7931.92
2	105	0.1	50	0.9903	7922.08
3	105	0.15	50	0.9819	7855.36
4	105	0.2	50	0.9824	7859.12
5	105	0.25	50	0.9818	7854.48
6	105	0.3	50	0.9830	7864.16
7	105	0.35	50	0.9797	7837.20
8	105	0.4	50	0.9739	7791.12
9	105	0.45	50	0.9706	7764.64
10	105	0.5	50	0.9586	7668.56
11	105	0.55	50	0.9492	7593.36
12	105	0.6	50	0.9413	7530.08

**Table A11:** Manufacturing parameters and experimental data adapted from [25]. Complementing relevant information and assumptions are the following; material: Ti6Al4V, theoretical density *ρ_th_* = 4220 kg/m^3^, heat conductivity *κ* = 6.4 W/mK and specific heat capacity *C_p_* = 546 J/kgK.

ID	*P* (W)	*v* (m/s)	*h* (μm)	Relative Density	Bulk Density (kg/m^3^)
1	175	0.2	100	0.9661	4077.03
2	175	0.3	100	0.9752	4115.18
3	175	0.4	100	0.9943	4195.90
4	175	0.5	100	0.9995	4218.02
5	175	0.6	100	0.9989	4215.27
6	175	0.7	100	0.9983	4212.78
7	175	0.8	100	0.9971	4207.89
8	175	0.9	100	0.9964	4204.81
9	175	1	100	0.9946	4197.30
10	175	1.1	100	0.9931	4191.05
11	100	0.6	100	0.9910	4181.89
12	111	0.6	100	0.9962	4203.92
13	122	0.6	100	0.9986	4213.88
14	133	0.6	100	0.9989	4215.40
15	144	0.6	100	0.9989	4215.15
16	155	0.6	100	0.9990	4215.74
17	166	0.6	100	0.9991	4216.33
18	177	0.6	100	0.9987	4214.39
19	188	0.6	100	0.9987	4214.30
20	200	0.6	100	0.9962	4203.92

**Table A12:** Manufacturing parameters and experimental data adapted from [26]. Complementing relevant information and assumptions are the following; material: Ti6Al4V, theoretical density *ρ_th_* = 4220 kg/m^3^, heat conductivity *κ* = 6.4 W/mK and specific heat capacity *C_p_* = 546 J/kgK.

ID	*P* (W)	*v* (m/s)	*h* (μm)	Relative Density	Bulk Density (kg/m^3^)
1	50	0.5	100	0.7764	3276.53
2	100	0.5	100	0.9959	4202.49
3	150	0.5	100	0.9160	3865.31
4	195	0.5	100	0.9074	3829.31
5	100	0.75	100	0.9362	3950.93
6	150	0.75	100	0.9959	4202.49
7	195	0.75	100	0.9778	4126.44
8	100	1	100	0.7976	3366.00
9	150	1	100	0.9970	4207.13
10	195	1	100	0.9982	4212.40
11	100	1.2	100	0.7465	3150.31
12	150	1.2	100	0.9459	3991.82
13	195	1.2	100	0.9989	4215.32

**Table A13:** Manufacturing parameters and experimental data adapted from [27]. Complementing relevant information and assumptions are the following; material: Ti6Al4V, heat conductivity *κ* = 6.4 W/mK and specific heat capacity *C_p_* = 546 J/kgK.

ID	*P* (W)	*v* (m/s)	*h* (μm)	Bulk Density (kg/m^3^)
1	75	1	77	4378.00
2	75	0.8	77	4392.25
3	75	0.6	77	4378.75
4	75	0.4	77	4378.50
5	75	0.3	77	4333.00
6	75	0.2	77	4297.50
7	75	0.15	77	4317.50

**Table A14:** Manufacturing parameters and experimental data adapted from [28]. Complementing relevant information and assumptions are the following; material: Ti26Nb, theoretical density *ρ_th_* = 6150 kg/m^3^, heat conductivity *κ* = 31.4 W/mK and specific heat capacity *C_p_* = 417 J/kgK.

ID	*P* (W)	*v* (m/s)	*h* (μm)	Relative Density	Bulk Density (kg/m^3^)
1	120	1.750	100	0.8031	4937.68
2	120	1.000	60	0.8749	5379.01
3	120	0.389	80	0.9498	5839.46
4	220	1.300	40	0.9940	6111.71
5	360	0.813	40	0.9843	6051.82
6	220	0.400	20	0.9916	6096.46
7	220	0.625	10	0.9851	6056.49

**Table A15:** Manufacturing parameters and experimental data adapted from [29]. Complementing relevant information and assumptions are the following; material: TiSiC, theoretical density *ρ_th_* = 4110 kg/m^3^, heat conductivity *κ* = 14.9 W/mK and specific heat capacity *C_p_* = 538 J/kgK.

ID	*P* (W)	*v* (m/s)	*h* (μm)	Relative Density	Bulk Density (kg/m^3^)
1	200	0.8	100	0.7100	2918.10
2	240	0.8	100	0.8200	3370.20
3	280	0.8	100	0.8430	3464.73
4	320	0.8	100	0.8380	3444.18
5	320	0.4	100	0.9230	3793.53
6	320	0.6	100	0.9260	3805.86
7	320	1	100	0.8670	3563.37
8	320	1.2	100	0.8100	3329.10
9	360	0.8	100	0.9000	3699.00

**Table A16:** Manufacturing parameters and experimental data, adapted from [30]. Complementing relevant information and assumptions are the following; material: W, theoretical density *ρ_th_* = 19,280 kg/m^3^, heat conductivity *κ* = 173 W/mK and specific heat capacity *C_p_* = 134 J/kgK.

ID	*P* (W)	*v* (m/s)	*h* (μm)	Relative Density	Bulk Density (kg/m^3^)
1	90	0.2	30	0.66	12,724.80
2	90	0.4	30	0.70	13,496.00
3	90	0.6	30	0.65	12,532.00
4	90	0.8	30	0.65	12,532.00
5	90	1	30	0.62	11,953.60
6	90	1.2	30	0.61	11,760.80
7	90	1.4	30	0.59	11,375.20
8	90	0.2	15	0.75	14,460.00
9	90	0.4	15	0.70	13,496.00
10	90	0.6	15	0.66	12,724.80
11	90	0.8	15	0.65	12,532.00
12	90	1	15	0.65	12,532.00
13	90	1.2	15	0.60	11,568.00
14	90	1.4	15	0.63	12146.40

**Table A17:** Manufacturing parameters and experimental data, adapted from [31]. Complementing relevant information and assumptions are the following; material: Cu, theoretical density *ρ_th_* = 8940 kg/m^3^, heat conductivity *κ* = 385 W/mK and specific heat capacity *C_p_* = 390 J/kgK.

ID	*P* (W)	*v* (m/s)	*h* (μm)	Relative Density	Bulk Density (kg/m^3^)
1	800	0.2	70	0.9617	8597.96
2	800	0.3	70	0.9874	8827.36
3	800	0.4	70	0.9940	8886.00
4	800	0.5	70	0.9865	8819.13
5	800	0.6	70	0.9843	8799.82
6	800	0.2	90	0.9820	8779.35
7	800	0.3	90	0.9846	8802.06
8	800	0.4	90	0.9869	8823.15
9	600	0.2	70	0.9812	8771.75
10	600	0.3	70	0.9871	8824.23
11	600	0.4	70	0.9840	8796.69
12	600	0.2	90	0.9847	8803.40
13	600	0.3	90	0.9869	8822.89
